# Human bone marrow mesenchymal stem cell-derived extracellular vesicles attenuate neuroinflammation evoked by focal brain injury in rats

**DOI:** 10.1186/s12974-019-1602-5

**Published:** 2019-11-13

**Authors:** Sylwia Dabrowska, Anna Andrzejewska, Damian Strzemecki, Maurizio Muraca, Miroslaw Janowski, Barbara Lukomska

**Affiliations:** 1 NeuroRepair Department, Mossakowski Medical Research Centre, PAS, 5 Pawinskiego Street, 02-106 Warsaw, Poland; 2 Department of Experimental Pharmacology, Mossakowski Medical Research Centre, PAS, 5 Pawinskiego Street, 02-106 Warsaw, Poland; 3 Department of Women’s and Children’s Health, University of Padua, Via Giustiniani 3, 35128 Padua, Italy

**Keywords:** Mesenchymal stem cells, Extracellular vesicles, Ischemic brain injury, Stroke, Intra-arterial transplantation, Immune response

## Abstract

**Background:**

Ischemic stroke is the major cause of long-term severe disability and death in aged population. Cell death in the infarcted region of the brain induces immune reaction leading to further progression of tissue damage. Immunomodulatory function of mesenchymal stem cells (MSCs) has been shown in multiple preclinical studies; however, it has not been successfully translated to a routine clinical practice due to logistical, economical, regulatory, and intellectual property obstacles. It has been recently demonstrated that therapeutic effect of intravenously administered MSCs can be recapitulated by extracellular vesicles (EVs) derived from them. However, in contrast to MSCs, EVs were not capable to decrease stroke-induced neuroinflammation. Therefore, the aim of the study was to investigate if intra-arterial delivery of MSC-derived EVs will have stronger impact on focal brain injury-induced neuroinflammation, which mimics ischemic stroke, and how it compares to MSCs.

**Methods:**

The studies were performed in adult male Wistar rats with focal brain injury induced by injection of 1 μl of 50 nmol ouabain into the right hemisphere. Two days after brain insult, 5 × 10^5^ human bone marrow MSCs (hBM-MSCs) labeled with Molday ION or 1.3 × 10^9^ EVs stained with PKH26 were intra-arterially injected into the right hemisphere under real-time MRI guidance. At days 1, 3, and 7 post-transplantation, the rats were decapitated, the brains were removed, and the presence of donor cells or EVs was analyzed. The cellular immune response in host brain was evaluated immunohistochemically, and humoral factors were measured by multiplex immunoassay.

**Results:**

hBM-MSCs and EVs transplanted intra-arterially were observed in the rat ipsilateral hemisphere, near the ischemic region. Immunohistochemical analysis of brain tissue showed that injection of hBM-MSCs or EVs leads to the decrease of cell activation by ischemic injury, i.e., astrocytes, microglia, and infiltrating leucocytes, including T cytotoxic cells. Furthermore, we observed significant decrease of pro-inflammatory cytokines and chemokines after hBM-MSC or EV infusion comparing with non-treated rats with focal brain injury.

**Conclusions:**

Intra-arterially injected EVs attenuated neuroinflammation evoked by focal brain injury, which mimics ischemic stroke, and this effect was comparable to intra-arterial hBM-MSC transplantation. Thus, intra-arterial injection of EVs might be an attractive therapeutic approach, which obviates MSC-related obstacles.

## Background

Stroke is the third cause of death in people after 60 years old in the developed countries and the main cause of disability. Every year, 15 millions of people worldwide suffer from stroke, 5 million of whom die and 5 million stay permanently disabled [1]. According to available data, therapy for people after stroke uses from 3% to 7% of total healthcare expenditures in the developed countries [2]. Ischemic stroke represents 87% of all stroke cases and typically originates from obstruction of oxygen and nutrient delivery to the brain due to intravascular clot formation [1]. Cell death in the ischemic region results in production of DAMPs (damage-associated molecular patterns) and induction of the local immune response in the brain. In the first few minutes after ischemic stroke, astrocytes and microglial cells are activated, which lead to production of pro-inflammatory cytokines, blood-brain barrier (BBB) injury, and progression of tissue damage [3]. Local inflammatory response activates endothelial cells, which facilitate infiltration of leucocytes to the nervous tissue and to further increase the damaged region in the brain [4]. On the other hand, the switch of astrocytes and microglia/macrophages’ phenotype from pro-inflammatory to anti-inflammatory may lead to production of anti-inflammatory cytokines and activation of T regulatory lymphocytes which have neuroprotective effect on injured tissue. Thus, there is a need to find a therapy which will modulate the immune reaction and limit the damage in the region injured by ischemia.

Many studies have shown that mesenchymal stem cells have immunomodulatory properties and can be potentially used in central nervous system therapies including ischemic stroke [5–8]. The past decade has seen an explosion of research directed toward better understanding of the mechanisms of MSC function during rescue of injured organs and tissues. On the contrary to the initial assumption, MSCs may not primary exert their functions in a cellular but rather in a paracrine manner. Recent studies suggest that the signals responsible for these therapeutic effects are at least partially linked to the production of extracellular vesicles (EVs).

The discovery of extracellular vesicles, naturally released by almost all cells, through which protein-, lipid-, and nucleic acid-based regulatory factors are transferred is a great promise for ischemic stroke treatment and could be an alternative for using stem cells. Despite the positive effects of stem cells in the models of experimental stroke, some researchers have demonstrated the risk of their application, including graft rejection after allogeneic transplantation [9], the possibility of small blood vessel obstruction [10], or tumor transformation [11]. Extracellular vesicles derived from stem cells do not have limits such as graft rejection, tumor formation, or small blood vessels’ blockage. Moreover, EVs have the ability to cross blood-brain barrier [12] and show greater stability during storage, and the procedure of their transplantation is easier than cells. Until now, the secretome of different stem cells has been shown to be effective in promoting neuroprotection and recovery in cell and animal models of central nervous system (CNS) disorders.

Previously, the characteristics of EVs derived from human MSCs were examined by us in vitro [13]. It has been recently demonstrated that therapeutic effect of intravenously administered MSCs can be recapitulated by extracellular vesicles derived from them. However, in contrast to MSCs, EVs were not capable to decrease stroke-induced neuroinflammation. Therefore, the aim of the study was to investigate if intra-arterial delivery of MSC-derived EVs will have more impact on focal brain injury-induced neuroinflammation, which mimics ischemic stroke, and how it compares to MSCs.

Using the focal brain injury model, EVs or MSCs were transplanted intra-arterially into adult Wistar rats and the grafts have been analyzed in the host brain. Moreover, the cellular immune response in the brain of transplant recipients was evaluated by immunohistochemical staining and the cytokine and chemokine levels were measured by multiplex immunoassay.

## Methods

### Cell culture

Commercially available human bone marrow mesenchymal stem cells (hBM-MSCs) (Lonza Inc., Walkersville, MD, USA) were plated in 75 cm^2^ polystyrene tissue culture flasks (Thermo Scientific) at a density of 5 × 10^3^cells/cm^2^ with 10 ml of Mesenchymal Stem Cell Growth Medium (MSCGM™, Bullet Kit, Lonza). Cultures were incubated at 37 °C in a humidified atmosphere containing 5% CO_2_. Cells were subsequently maintained in MSCGM™ medium and passaged at 80% confluence in a ratio 1:3 in trypsin/EDTA solution (Gibco, Life Technologies) with the culture medium changed thrice a week.

### EV isolation from hBM-MSCs

The isolation of EVs was performed from conditioning media of non-labeled hBM-MSCs. 5 × 10^6^ of hBM-MSCs (passages 4–6) were cultured in 75 cm^2^ polystyrene tissue flasks to reach 50–60% confluence, then the culture medium was changed, and the cells were incubated for additional 48–72 h to the confluence of 70–80%. Cell culture supernatants were collected and centrifuged at 200*g* for 10 min, then at 500*g* for 10 min at 4 °C, aliquoted and frozen at − 70 °C for further use. In order to isolate EVs, hBM-MSC culture supernatants were thawed, spun down at 2000*g* for 20 min to remove cellular debris, and then centrifuged at 100,000*g* for 75 min at 4 °C using a Thermo Scientific Type 865 Fixed Angle Rotor. The pellets were washed with deionized phosphate-buffered saline (DPBS) and subjected to an additional centrifugation at 100,000*g* for 75 min at 4 °C using a Thermo Scientific Type 865 Fixed Angle Rotor. Then, the supernatant was discarded and the pellet was re-suspended in 1 ml of DPBS and stored at − 70 °C until needed.

### Labeling of hBM-MSCs

The labeling of cells with Molday ION consisted of superparamagnetic iron oxide nanoparticles (SPIO) and rhodamine B purchased from BioPAL (Worcester, USA) was performed as previously described by us. Briefly, 100 μl of Molday ION was added to the 5 × 10^5^ hBM-MSCs cultured in 10 ml Mesenchymal Stem Cell Growth Medium and incubated over 16 h at 37 °C in a humidified atmosphere containing 5% CO_2_. After that, medium with label was removed, cells were washed with phosphate-buffered saline (PBS), fresh medium was added, and cells were cultured 48 or 72 h.

### Labeling of EVs using PKH26

EVs isolated from non-labeled hBM-MSCs were tagged with PKH26 (Red Fluorescent Cell Linker Kits MINI26; Sigma-Aldrich Co., St Louis, MO, USA) at room temperature (RT) for 5 min in the dark and blocked with fetal bovine serum (FBS), according to manufacturer’s instructions. The unincorporated labels were removed by EV centrifugation at 100,000×*g* for 75 min at 4 °C using a Thermo Scientific T-865 Fixed Angle Rotor Thermo Scientific Sorvall WX Ultracentrifuge Series. EVs were washed with DPBS and subjected to additional centrifugations. Then, the pellet was re-suspended in 1 ml DPBS for further use.

### NanoSight particle tracking analysis of EVs isolated from hBM-MSCs

The size and concentration of EVs were analyzed using NanoSight NS300 system (Malvern, UK), configured with sCMOS camera and blue 488 nm laser. For NanoSight analysis, extracellular vesicles were diluted in 1 ml DPBS and collected and analyzed by NanoSight tracking analysis (NTA) software version 3.2. Each of EV samples from the different isolations was recorded three times for 60 s at constant temperature 23 °C creating three replicable histograms which were averaged.

### Western blot analysis

hBM-MSC or EV pellets were re-suspended in RIPA lysis buffer. Protein concentrations were determined using a Bio-Rad DC™protein assay kit (Bio-Rad) in the supernatant as well as in the pellet solution. Samples of the pellet were ran on 10–15% SDS-PAGE gels and transferred onto nitrocellulose membranes (Amersham Bioscience). After blocking, membranes were probed with calnexin anti-body (Millipore) and then incubated with horseradish peroxidase-conjugated secondary IgG antibodies (Sigma-Aldrich). Immunoblot signals were visualized using ECL chemiluminescence kit (GE Healthcare Life Sciences). The β-actin antibody was used as an internal control.

### Rat model of focal brain injury

Adult male Wistar rats, weighing 250 g and housed in cages with a 12-h light-dark cycle and free access to food and water, were used in all experiments. Rats were anesthetized with a mix of Bioketan (Vetoquinol; 53.6 mg/kg) and Domitor (Orion Pharma; 0.4 mg/kg) by i.p. injection and placed in a stereotaxic apparatus (Stoelting). The model of brain infarct was performed as previously described by us [14]. Briefly, a burr hole was drilled in the skull and the needle (length 15 mm, gage 33), connected to a 10-μl syringe (Hamilton, Switzerland), was lowered into the right striatum (coordinates A 0.5, L 3.8, D 4.7 mm). Then, 1 μl of 5 nmol ouabain (Sigma, Poland) was injected into the brain at a speed of 1 μl/min using a microinfusion pump (Stoelting, USA). The needle was then withdrawn, and the skin was closed with a suture. After the procedure, all animals were treated with an antibiotic (Baytril; Bayer; 0.4 mg/ml) and an analgesic (Rycarfa; Krka; 5 mg/ml).

### Intra-arterial transplantation of hBM-MSCs or EVs

Intra-arterial infusion of hBM-MSCs or EVs was performed 48 h after the induction of focal brain injury as previously described by us. Briefly, under general anesthesia (2% isoflurane), the common carotid artery (CCA), the external carotid artery (ECA), and the internal carotid artery (ICA) were exposed, the occipital artery branching off the ECA was coagulated, and the pterygopalatine artery branching off the ICA was ligated, as well as the proximal segments of the ECA and CCA. Then, the vascular clip (FT 180T, Aesculap, Center Valley, PA, USA) was applied to the ICA proximal to the pterygopalatine artery to prevent backflow, the incision into the CCA was performed distal to the ligature, and the catheter was inserted into the CCA, followed by the suture tightening on the artery over the catheter to prevent bleeding. Then, the clip was removed, the animal was placed inside the gantry of the MR scanner, and 5 × 10^5^ hBM-MSCs labeled with Molday ION or 1.3 × 10^9^ EVs stained with PKH26 were transplanted into the right internal carotid artery of Wistar rats in 1 ml of PBS at a safe speed of 0.2 ml/min.

### Magnetic resonance imaging and quantitative image analysis of hBM-MSCs

The infusion of hBM-MSCs was imaged with a 7-T Biospec 70/30 MR scanner (Bruker), with a transmit cylindrical radiofrequency coil (8.6 cm inner diameter) and a rat brain-dedicated, receive-only array coil (2 × 2 elements) positioned over the animal’s head. Directly before and after transplantation, animals were imaged using a T2 sequence (RARE, TR = 4000 ms, TE = 58.5 ms, TA = 2m 8s, FOV = 2.69/2.35, MTX = 256/128) to detect brain injury and with a T2* sequence (FLASH, TR = 500 ms, TE = 5 ms, FA = 40 deg, TA = 48 s, FOV = 2.69/2.35, MTX = 256/128) to detect injected cells.

### Brain tissue collection and preparation

One, three, or seven days after the intra-arterial transplantation of hBM-MSCs or EVs, rats were deeply anesthetized by intraperitoneal administration of Bioketan (53.6 mg/kg) and Domitor (0.4 mg/kg) and decapitated. The brains were isolated, immediately frozen on dry ice, and stored at − 70 °C. For immunohistochemical analysis, the brains were incubated at − 20 °C, cut on cryostat into coronal tissue sections 25 μm thick, placed on microscope slides, and frozen at − 70 °C. For cytokine expression detection, the brains were incubated at 4 °C, homogenized in RIPA lysis buffer containing 20 mM Tris-HCl (pH = 7.5), 150 mM NaCl, 1 mM PMSF, 0.05 Tween-20, and proteinase and phosphatase inhibitor cocktail (Sigma). The lysates were clarified by centrifugation at 11,500 rpm for 10 min at 4 °C, and the supernatants were collected and re-suspended in RIPA lysis buffer.

### Immunohistochemistry

The following antibodies (source and final dilution) were used for brain tissue staining: mouse monoclonal anti-human CD44 (Santa Cruz, 1:100), mouse monoclonal anti-human STEM121 (Cellartis, 1:100), mouse monoclonal anti-rat ED1 (Serotec, 1:100), rabbit monoclonal anti-rat glial fibrillary acidic protein (GFAP) (Dako, 1:200), mouse monoclonal anti-rat CD45RA (Serotec, 1:100), and mouse monoclonal anti-rat CD8 (Serotec, 1:300). Coronal cryostat sections of the brain (25 μm thick) were cut in serial order to create 10 series sections. Double fluorescent immunohistochemistry was performed. After blocking for unspecific reactivity, adjacent series of sections were stained for a specific cell-lineage marker. Tissue sections were rinsed in PBS and then incubated in 10% goat serum in PBS containing 0.25% Triton X-100 and 0.1% BSA for 60 min in RT. Next, the sections were washed with PBS and incubated with primary antibodies overnight at 4 °C. The following day, tissue sections underwent the washing procedure, and the primary antibodies were revealed by applying appropriate secondary antibodies (Alexa Fluor, 1:500) for 60 min at room temperature and in the dark. Nuclei were subsequently labeled with the fluorescent dye 5 μM Hoechst 33258 (Life Technologies). Labeling was verified using a confocal laser scanning microscope (LSM 780, Carl Zeiss, Germany) and Cell Observer SD (Carl Zeiss, Germany) using × 20 or × 40 objectives. A helium-neon laser (543 nm) was utilized in the excitation of Alexa Fluor 546, while an argon laser (488 nm) was applied in the excitation of FITC. ZEN software was used for quantitative analysis of immunoreactivity in all sections. Six animals per group were analyzed. Images from two sections per animal were taken, and the number of positive-labeled cells was assessed.

### Determination of cytokine expression in brain extracts

Concentrations of cytokines and chemokines were measured in extracts from brain tissues using the BioPlexPro Rat Cytokine 23-plex Assay (BioRad) according to the manufacturer’s instructions. The cytokines and chemokines analyzed included interleukin-1α (IL-1α), interleukin-1 β (IL-1β), interleukin-6 (IL-6), transforming growth factor-β2 (TGF-β2), chemokine C-X-C motif ligand-1 (CXCL1), monocyte chemoattractant protein-1 (MCP-1), macrophage inflammatory protein-1α (MIP-1α), and macrophage inflammatory protein-3α (MIP-3α). The median fluorescence intensity plates were assayed on a Bio-Plex®200 Luminex system with Bio-Plex Manager 5.0 software. The five-parameter logistic method was applied to estimate cytokine/chemokine concentrations in brain homogenates.

### Statistical analysis

The results were shown as mean ± SD. The statistical analysis was performed using the one-way ANOVA and the Tukey test. In all calculations, the program GraphPad Prism 3.0 was used. The significance level less than 0.05 was considered as statistically significant.

## Results

### Analysis of EVs isolated from hBM-MSCs

**Fig. 1:**
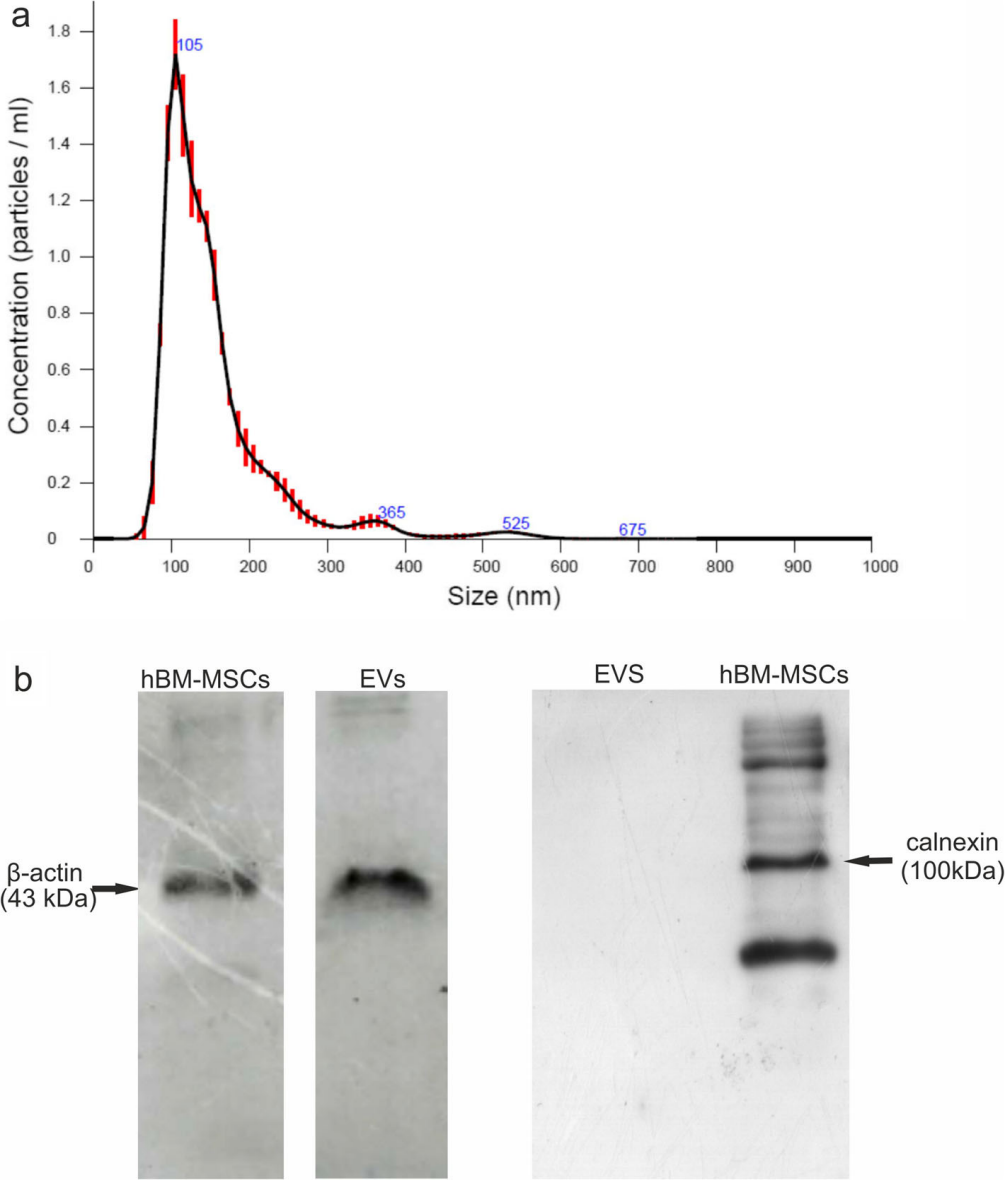
**a** NanoSight analysis measurements of sizes and concentrations of extracellular vesicles (EVs) secreted by human bone marrow-derived mesenchymal stem cells (hBM-MSCs). Representative graph shows the results of particle concentration and their size measurements. NTA analysis of this sample revealed two populations of EVs with peak diameter of 105 and 365 nm. **b** The Western blot analysis of EVs isolated from hBM-MSCs. EVs do not express calnexin, which is the membrane marker expressed on hBM-MSCs

NanoSight particle tracking analysis revealed that EVs derived from hBM-MSCs represented heterogeneous population consisted of exosomes/smaller microvesicles with the average peak at 111.7 ± 11.5 nm and larger microvesicles with the average peak at 398.3 ± 49.3 nm (Fig. 1a). The average concentration of EVs isolated from 5 × 10^6^ cells was 1.3 × 10^9^ ± 1.8 × 10^7^/ml which corresponded to 110.2 ± 24.0 particles/frame and 134.9 ± 37.6 centers/frame. The expression of specific proteins was assessed in EVs and hBM-MSCs by Western blot confirming the presence of cell-associated calnexin, a calcium-binding, ER-associated protein in hBM-MSCs but the absence of calnexin in EVs (Fig. 1b).

### MRI of hBM-MSCs after intra-arterial transplantation

**Fig. 2:**
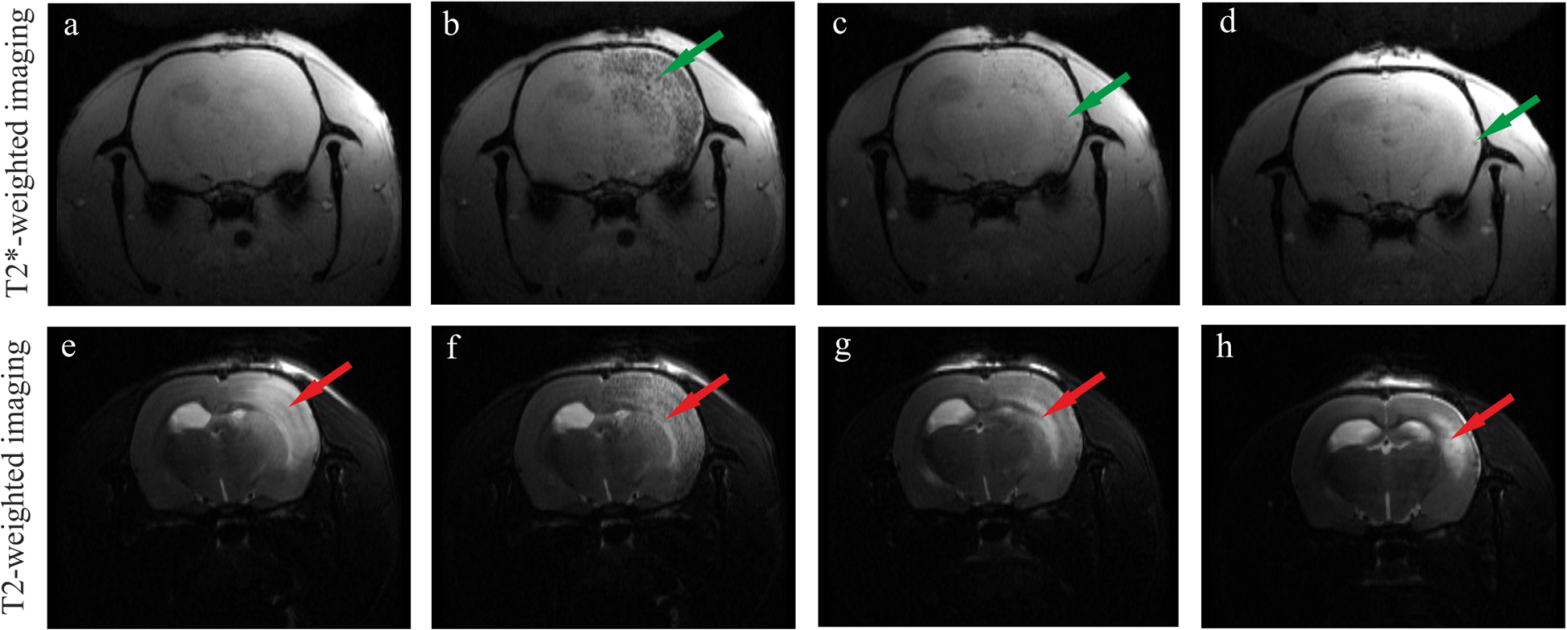
Representative MR images of rat brains 2 days after focal injury insult. T2* images show the hypointensive signal coming from transplanted cells before (**a**), few minutes (**b**), 24 h (**c**), and 7 days (**d**) after intra-arterially injected hBM-MSCs labeled with Molday ION whereas the T2 images show the localization of injured brain region before (**e**), few minutes (**f**), 24 h (**g**), and 7 days (**h**) after intra-arterial hBM-MSC injection

The MRI analysis enables visualization of transplanted cells labeled with Molday ION as dark spots (Fig. 2b–d, green arrows). The region of injured brain is seen as brighter area (Fig. 2e–h, red arrows). The results of our studies showed that hBM-MSCs stained with Molday ION injected into the right internal carotid artery were routed into focal injury of the rat right hemisphere (Fig. 2b, f). After 24 h, the signal generated from transplanted cells significantly decreased (Fig. 2c, g). Seven days after hBM-MSC intra-arterial infusion, only single dark spots were observed in some rats (Fig. 2d, h).

### Immunohistochemistry of hBM-MSCs transplanted into the right hemisphere

**Fig. 3:**
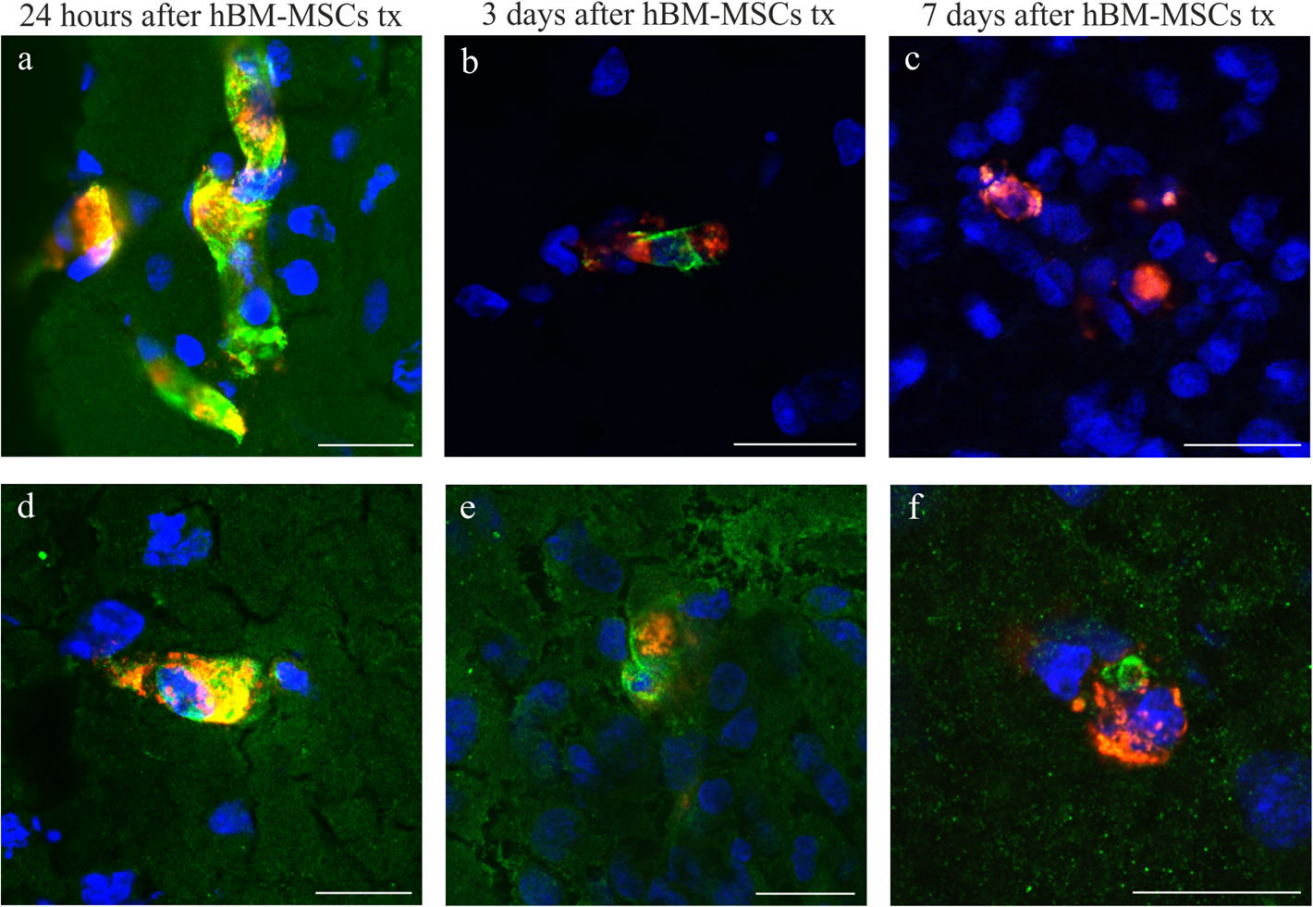
Human bone marrow mesenchymal stem cells (hBM-MSCs) visible in the ipsilateral hemisphere of focal injured rats 24 h, 3 days, and 7 days after intra-arterial cell infusion. Representative images of hBM-MSCs stained with Molday ION (red) and co-labeled with CD44 (**a**–**c**) or STEM-121 (**d**–**f**) markers (green). Scale bar 20 μm

To characterize hBM-MSCs present in the rat brain after their intra-arterial transplantation, immunohistochemical staining for human stem cell markers CD44 and STEM121 was performed. The analysis revealed that hBM-MSCs stained with Molday ION seen in the right hemisphere co-expressed CD44 marker 24 h (Fig. 3a), 3 days (Fig. 3b), and 7 days (Fig. 3c) after transplantation. Furthermore, hBM-MSCs labeled with Molday ION observed in the rat brain were positive for STEM121 24 h (Fig. 3d), 3 days (Fig. 3e), and 7 days (Fig. 3f) after their intra-arterial injection.

### The analysis of EV presence in the right hemisphere after intra-arterial injection

**Fig. 4:**
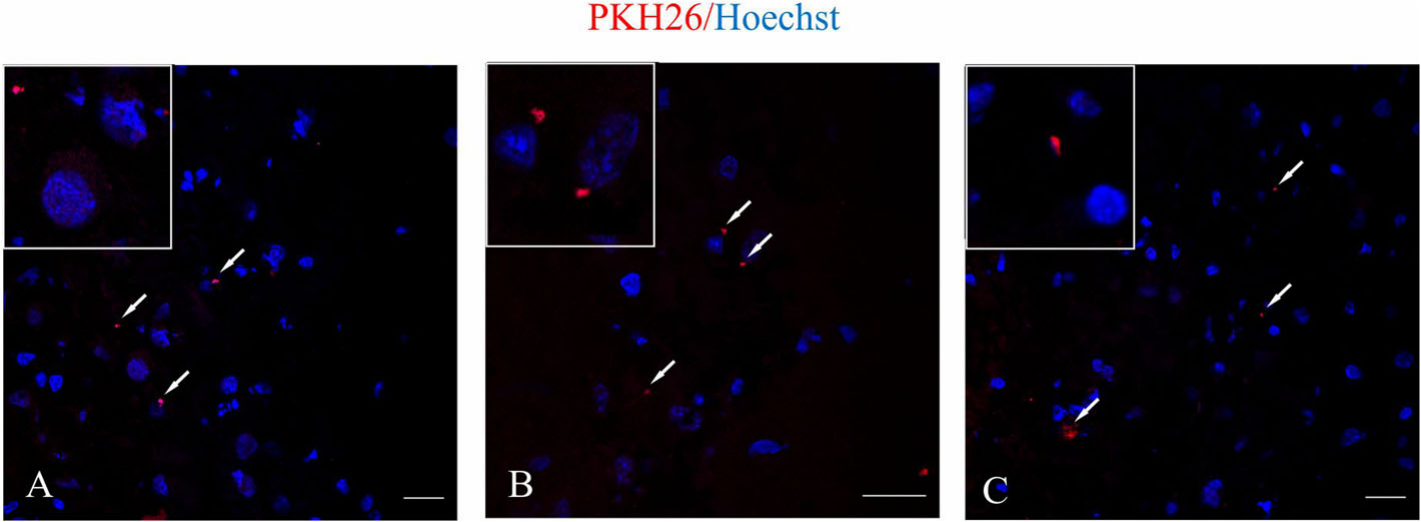
Extracellular vesicles (EVs) derived from human bone marrow mesenchymal cells (hBM-MSCs) enter the ipsilateral hemisphere of focal injured rats after intra-arterial infusion. Representative images of EVs labeled with PKH26 visible in the different regions of ipsilateral hemisphere of graft recipients 24 h after EV transplantation (**a-c**). Scale bar 10 μm

To detect EVs labeled with PKH26 in the rat brain after transplantation into the right internal carotid artery, the analysis in confocal microscope was performed. The study revealed that EVs stained with PKH26 were observed in the rat right hemisphere 24 h after intra-arterial transplantation (Fig. 4a–c).

### Characterization of immune cells in the rat brain

**Fig. 5:**
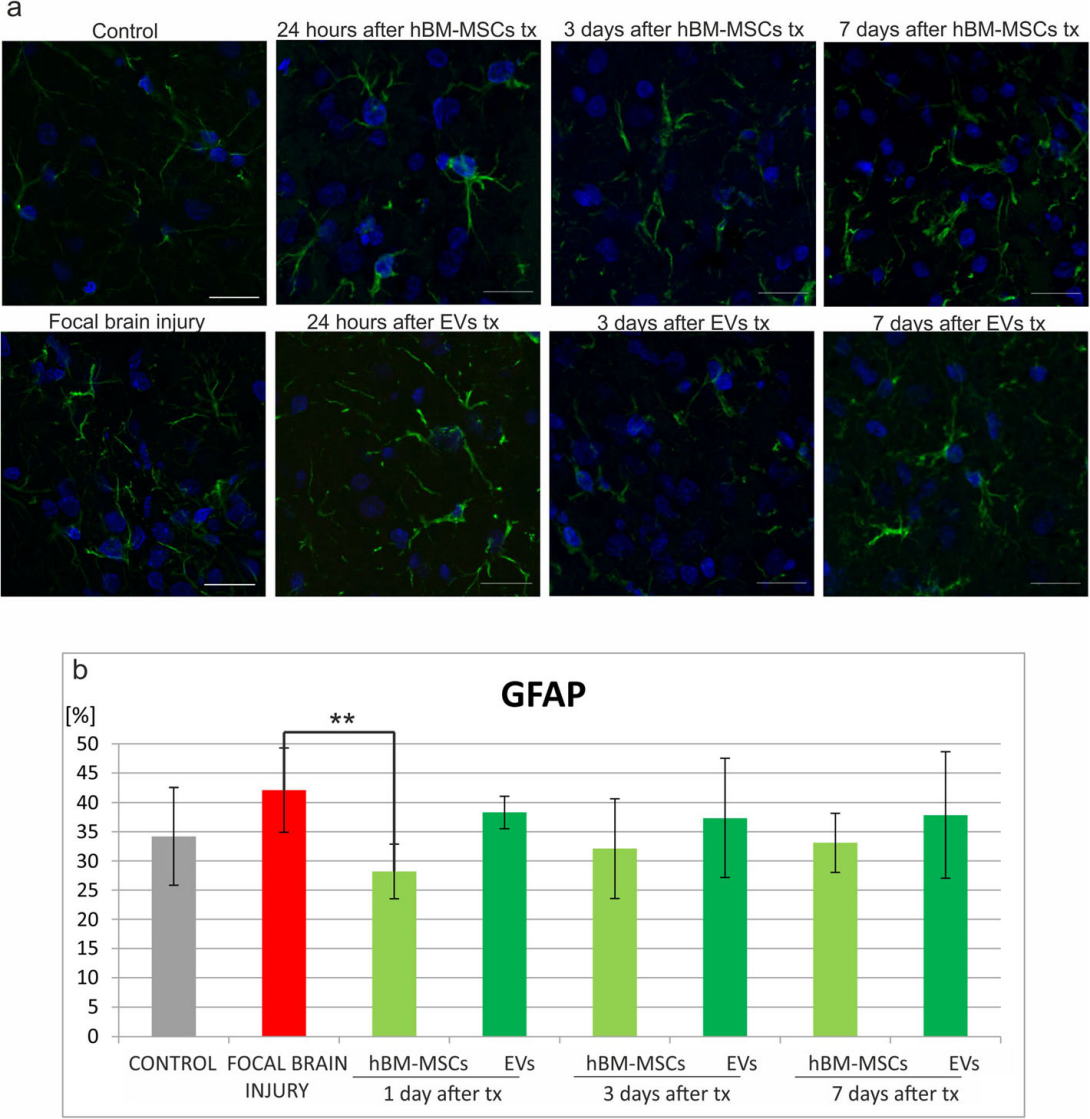
Human bone marrow mesenchymal stem cells (hBM-MSCs) or their extracellular vesicles (EVs) transplanted intra-arterially alleviate GFAP+ cells in the rat brain evoked by focal brain injury. **a** Representative images of GFAP staining in the rat brain of control rats, focal brain injured rats left intact, and those receiving hBM-MSC or EV transplantation. Scale bars 20 μm. **b** Quantitative analysis of the host cells positive for GFAP observed in the rat brains of focal brain injured rats 24 h, 3 days, and 7 days after hBM-MSC or EV transplantation versus untreated or control rats. All data reflect mean ± SD from 6 animals. *p < 0.1; **p < 0.01; ***p < 0.001; ****p < 0.0001

**Fig. 6:**
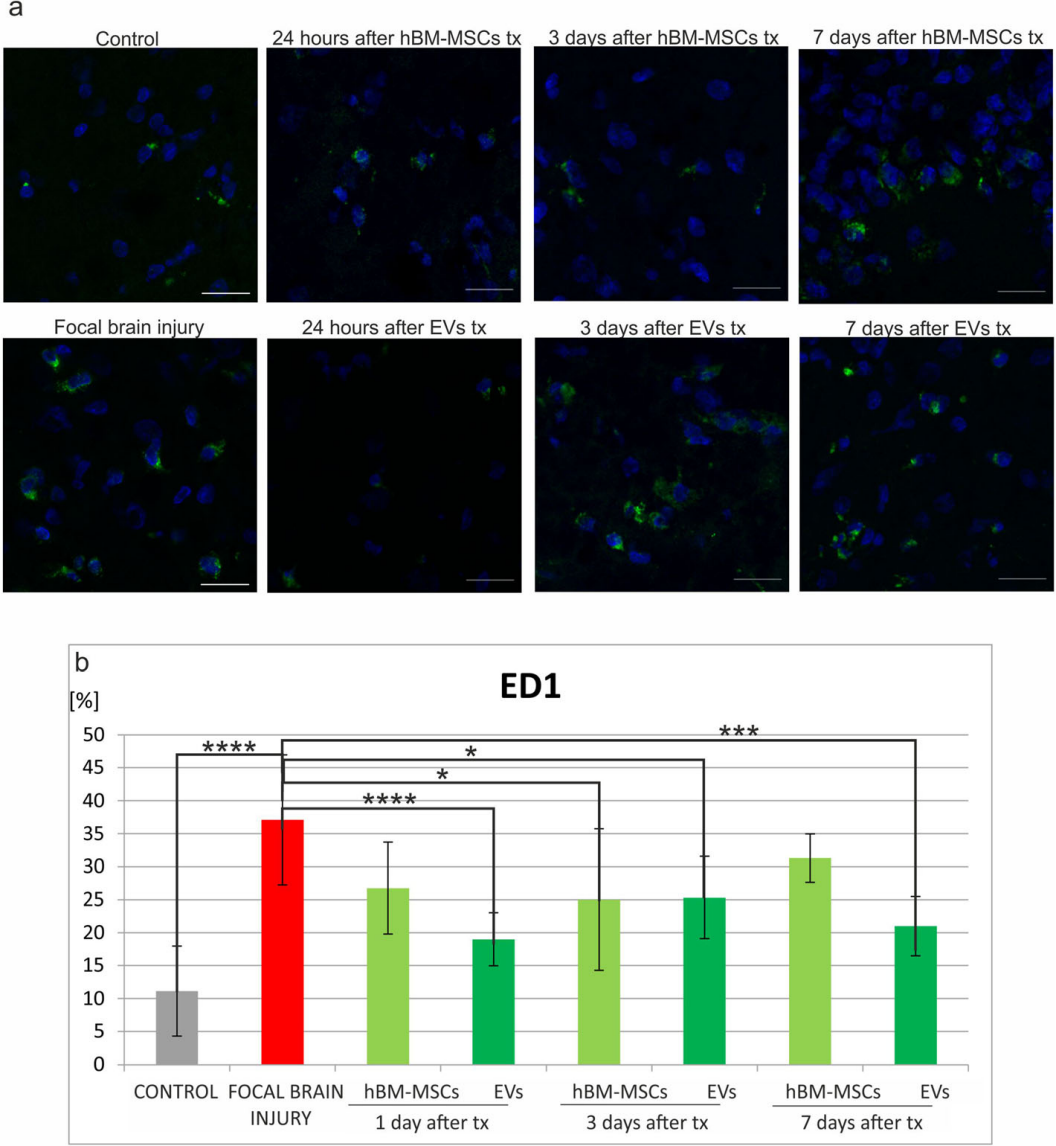
Human bone marrow mesenchymal stem cells (hBM-MSCs) or their extracellular vesicles (EVs) transplanted intra-arterially alleviate ED1+ cells in the rat brain evoked by focal brain injury. **a** Representative images of ED1 staining in the rat brain of control rats, focal brain injured rats left intact, and those receiving hBM-MSC or EV transplantation. Scale bars 20 μm. **b** Quantitative analysis of the host cells positive for ED1 observed in the rat brains of focal brain injured rats 24 h, 3 days, and 7 days after hBM-MSC or EV transplantation versus untreated or control rats. All data reflect mean ± SD from 6 animals. *p < 0.1; **p < 0.01; ***p < 0.001; ****p < 0.0001

**Fig. 7:**
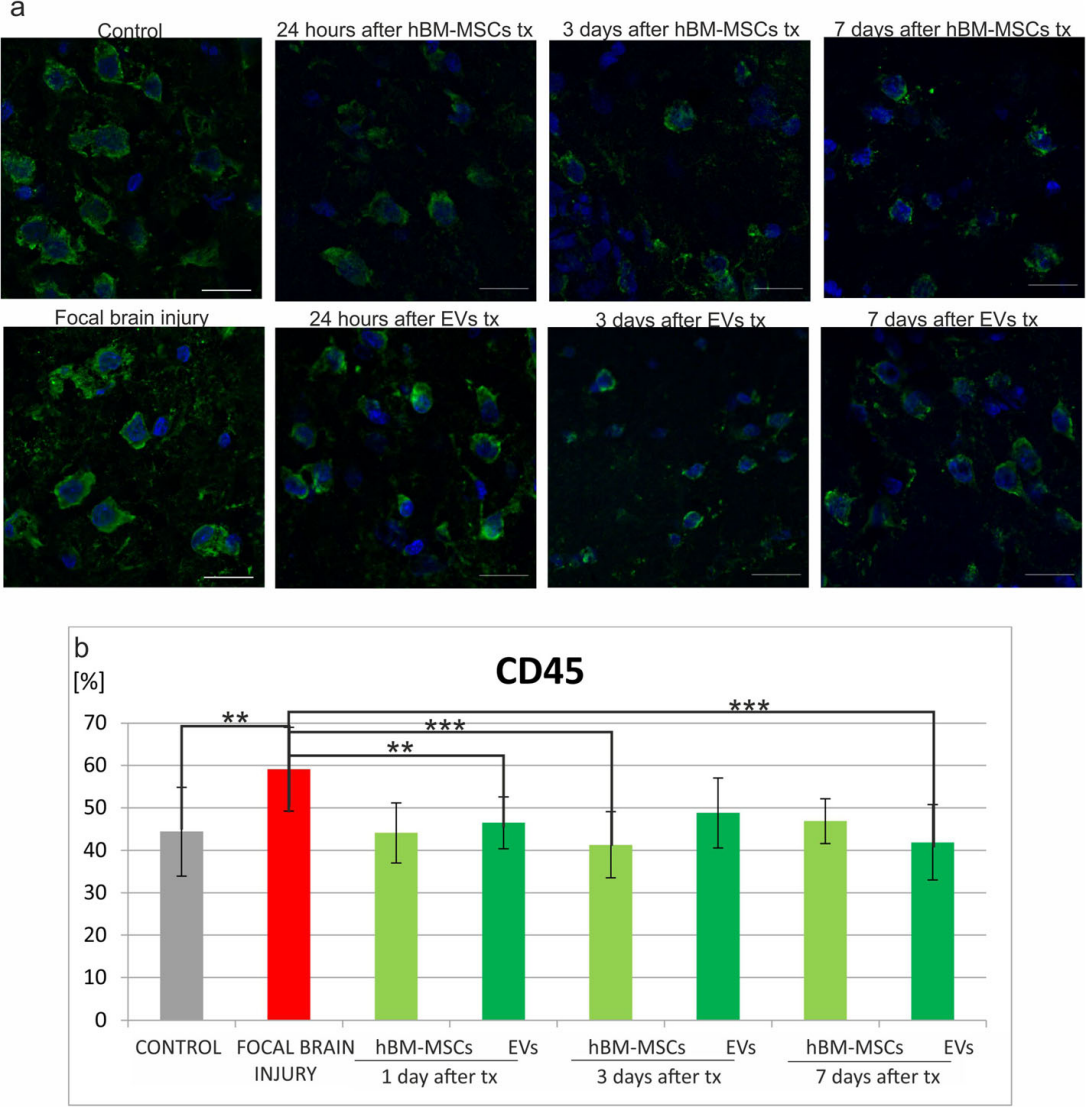
Human bone marrow mesenchymal stem cells (hBM-MSCs) or their extracellular vesicles (EVs) transplanted intra-arterially alleviate CD45RA+ cells in the rat brain evoked by focal brain injury. **a** Representative images of CD45RA staining in the rat brain of control rats, focal brain injured rats left intact, and those receiving hBM-MSC or EV transplantation. Scale bars 20 μm. **b** Quantitative analysis of the host cells positive for CD45RA observed in the rat brains of focal brain injured rats 24 h, 3 days, and 7 days after hBM-MSC or EV transplantation versus untreated or control rats. All data reflect mean ± SD from 6 animals. *p < 0.1; **p < 0.01; ***p < 0.001; ****p < 0.0001

**Fig. 8:**
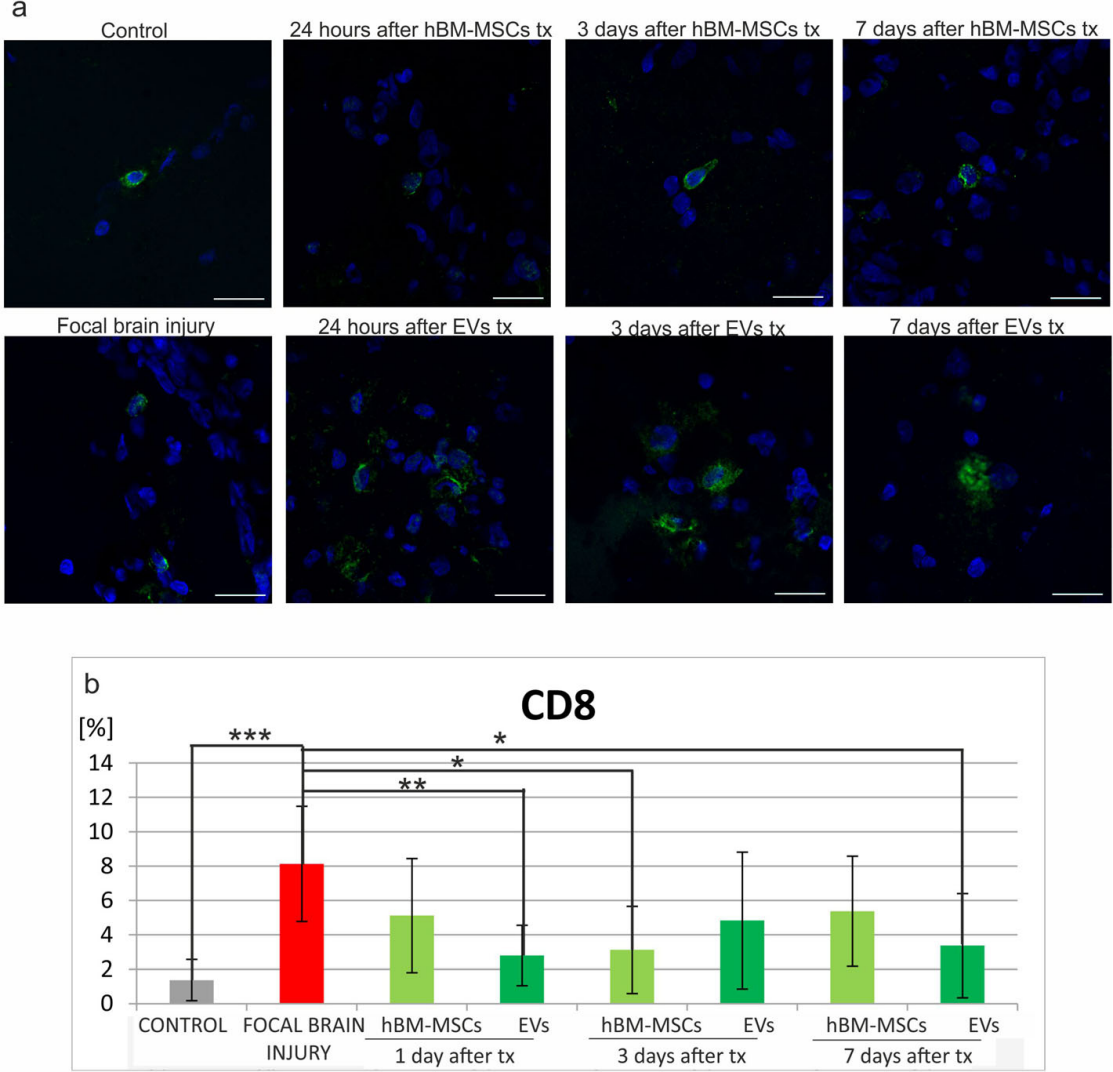
Human bone marrow mesenchymal stem cells (hBM-MSCs) or their extracellular vesicles (EVs) transplanted intra-arterially alleviate CD8+ cells in the rat brain evoked by focal brain injury. **a** Representative images of CD8 staining in the rat brain of control rats, focal brain injured rats left intact, and those receiving hBM-MSC or EV transplantation. Scale bars 20 μm. **b** Quantitative analysis of the host cells positive for CD8 observed in the rat brains of focal brain injured rats 24 h, 3 days, and 7 days after hBM-MSC or EV transplantation versus untreated or control rats. All data reflect mean ± SD from 6 animals. *p < 0.1; **p < 0.01; ***p < 0.001; ****p < 0.0001

The number of immune cells was counted in native control rats, in experimental animals with focal brain injury, and those which additionally received hBM-MSC or EV transplantation. Immunohistochemical analysis of the rat brain revealed that injection of ouabain into the rat striatum caused the increase of immune cells in the right hemisphere comparing to intact animals, such as microglial/macrophages ED1^+^ (Fig. 5b), astrocytes GFAP^+^ (Fig. 6b), leucocytes CD45RA^+^ (Fig. 7b), and T CD8^+^ lymphocytes (Fig. 8b).

Intra-arterial infusion of hBM-MSCs or EVs into the rat brain 48 h after induction of brain injury reduced the number of ED1^+^ microglia/macrophages in the rat brain. The effect was much stronger after extracellular vesicle than hBM-MSCs infusion (Fig. 5b). Transplantation of hBM-MSCs or EVs produced the reduction of astrocyte activation; however, statistically significant difference was noticed only 24 h after hBM-MSC infusion (Fig. 6b). This effect was accompanied by the decrease of leukocyte number (CD45RA^+^) after hBM-MSC or EV injection. Significant loss was observed 24 h and 7 days after EV transplantation (Fig. 7b). Among this population, the number of T lymphocytes expressing CD8 marker was found lower in rats receiving hBM-MSC or EV graft compared with the ischemic brain injured animals left without transplantation, although not in all time points of observation the differences were statistically significant (Fig. 8b).

### The analysis of cytokines and chemokines in the rat brain

**Fig. 9:**
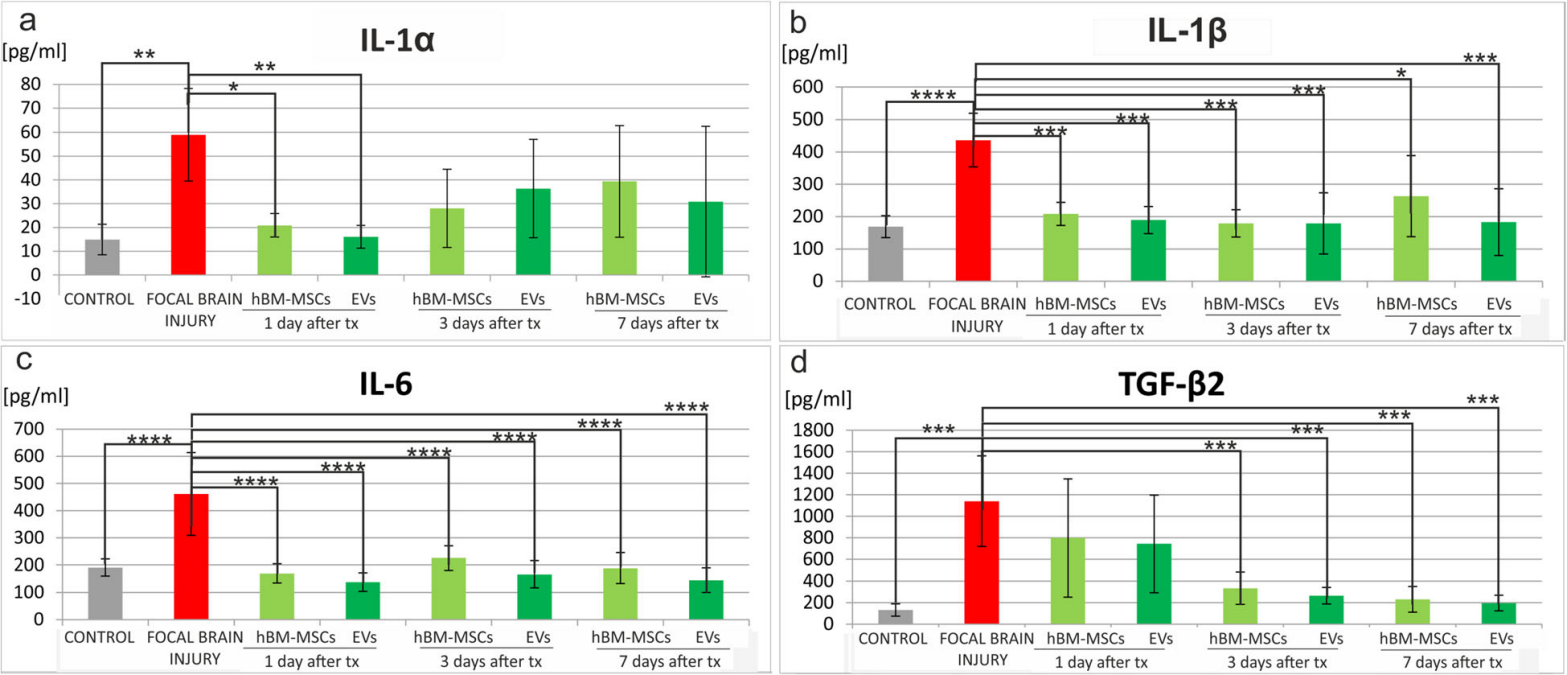
Human bone marrow mesenchymal stem cells (hBM-MSCs) or their extracellular vesicles (EVs) transplanted intra-arterially reduced pro-inflammatory cytokines observed in the rat brain after focal brain injury. Quantitation of IL-1α (**a**), IL-1β (**b**), IL-6 (**c**), and TGF-β2 (**d**) protein levels detected in the rat brains of focal brain injured rats 24 h, 3 days, and 7 days after hBM-MSC or EV transplantation versus untreated or control rats. All data reflect mean ± SD from 6 animals. *p < 0.1; **p < 0.01; ***p < 0.001; ****p < 0.0001

**Fig. 10:**
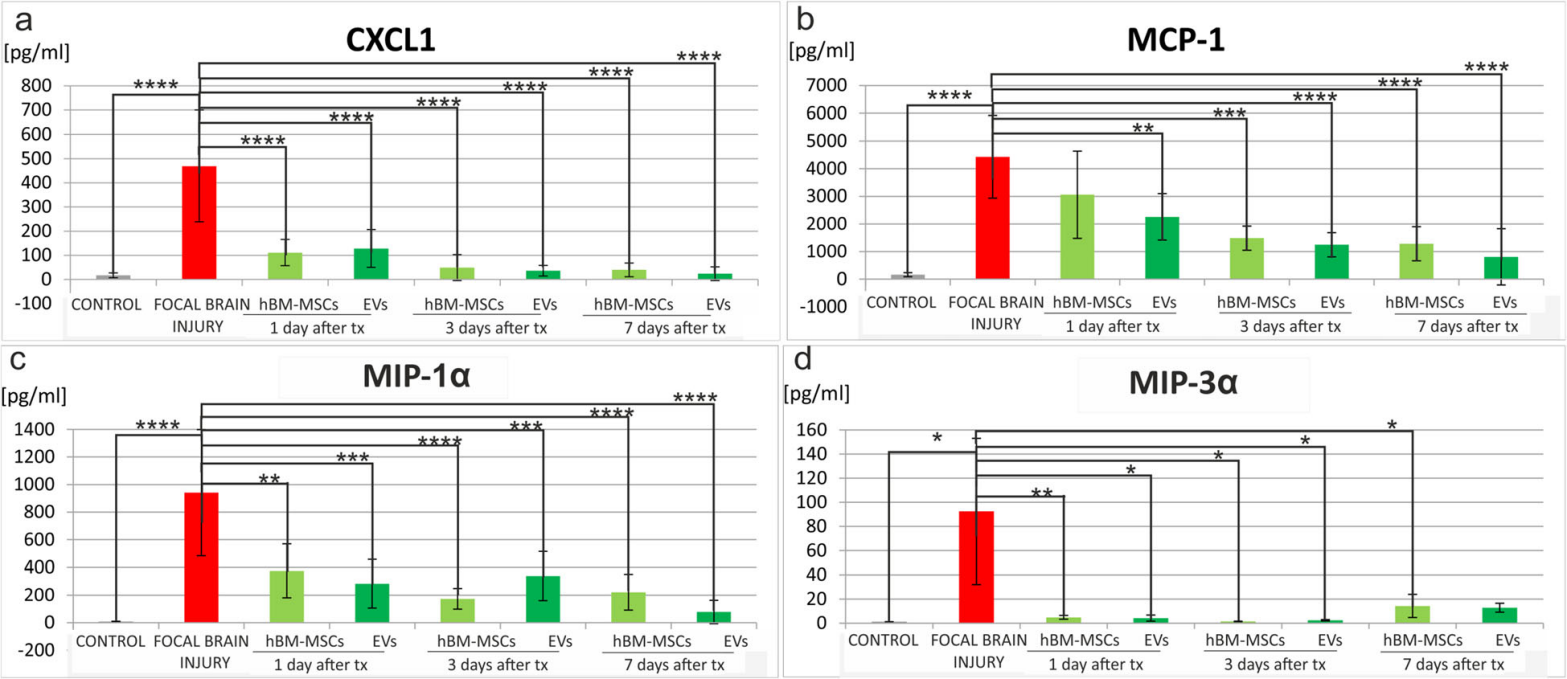
Human bone marrow mesenchymal stem cells (hBM-MSCs) or their extracellular vesicles (EVs) transplanted intra-arterially reduced the elevated level of chemokines noticed in the rat brain after focal brain injury. Quantitation of CXCL1 (**a**), MCP-1 (**b**), MIP-1α (**c**), and MIP-3α (**d**) protein levels detected in the rat brains of focal brain injured rats 24 h, 3 days, and 7 days after hBM-MSC or EV transplantation versus untreated or control rats. All data reflect mean ± SD from 6 animals. *p < 0.1; **p < 0.01; ***p < 0.001; ****p < 0.0001

The level of cytokines and chemokines was measured in the right hemisphere of control rats, rats with focal brain injury, and those with striatal insult accompanied with MSC or EV transplantation. The analysis using BioPlexPro Rat Cytokine 23-plex Assay (BioRad) revealed that ischemic brain injury caused statistically significant elevation of pro-inflammatory cytokines, such as IL-1α (*p* < 0.01) (Fig. 9a), IL-1β (*p* < 0.0001) (Fig. 9b, IL-6 (*p* < 0.0001) (Fig. 9c), and TGF-β2 (*p* < 0.001) (Fig. 9d), and chemokines, such as CXCL-1 (*p* < 0.0001) (Fig. 10a), MCP-1 (*p* < 0.0001) (Fig. 10b), MIP-1α (*p* < 0.0001) (Fig. 10c), and MIP-3α (*p* < 0.05) (Fig. 10d).

The expression of most of pro-inflammatory cytokines was reduced after hBM-MSC or EV injection. Infusion of hBM-MSCs or EVs led to the decrease of IL-1α; however, statistically significant difference was noticed 24 h after hBM-MSC (*p* < 0.05) and EV (*p* < 0.01) transplantation (Fig. 9a). The reduction of IL-1β was observed 24 h after hBM-MSC or EV infusion (*p* < 0.001); the statistically significant decrease was seen after 3 and 7 days of observation in rats receiving hBM-MSC or EV transplantation (Fig. 9b). Also, the level of IL-6 was significantly lower after injection of hBM-MSCs or EVs in each time point (*p* < 0.0001) (Fig. 9c). Concomitantly, the lower expression of TGF-β2 was detected after hBM-MSC or EV infusion; statistically significant difference was observed 3 and 7 days after cell or vesicle transplantation (*p* < 0.001) (Fig. 9d).

Transplantation of mesenchymal stem cells or extracellular vesicles resulted in the decrease of chemokines expression. The infusion of hBM-MSCs or EVs led to the reduction of CXCL1, highly statistically significant in each time point (*p* < 0.0001) (Fig. 10a). The difference in MCP-1 level was also statistically significant after hBM-MSC or EV infusion, which was observed stronger due to the longer time of observation after transplantation (Fig. 10b). We also noticed the strong reduction of the MIP-1α level after hBM-MSC or EV infusion in each time point (Fig. 10c). Moreover, the expression of MIP-3α decreased after stem cell or EV transplantation and its level was similar to this detected in control rats (Fig. 10d).

## Discussion

Stroke is accompanied with inflammatory and immune reactions which have been shown to be activated at each stage of disease from the early destructive events to the latest events of impaired brain tissue repair and vascular regeneration. Neuroinflammation has been suggested as an attractive treatment target in stroke, since it offers a broader therapeutic window in comparison to currently established thrombolytic approaches. Mesenchymal stem cells have recently emerged as promising candidates for cell-based therapy in neurological disorders. It might be that the extracellular vesicles isolated from MSCs which cross blood-brain barrier and easily migrate into the brain seem to be a promising alternative. In the current study, we highlighted the immunomodulation effect of human bone marrow-derived mesenchymal stem cells or extracellular vesicles released from these cells after their transplantation into focal brain injured rats. One of the critical questions in cell transplantation is about the route of graft delivery in terms of its efficacy and safety. The intravascular route of stem cell infusion has met the increasing interest because of less invasive procedure comparing to stereotactic injection. Unfortunately, the evaluation of cell distribution after intravenous infusion in neurologic disorders has shown that most of the cells are initially entrapped within the lungs and do not travel to the brain [15]. Recent studies have shown that an intra-arterial approach would be a more efficient way of cell delivery to CNS [16–19]. In neurological disorders, the transplantation procedure into internal carotid artery seems to be an effective way of cell injection into the brain. In the current study, we performed hBM-MSC or EV infusion into the right internal carotid artery ipsilateral to the focal rat brain insult.

The results of our experiments have proved that hBM-MSCs stained with Molday ION injected intra-arterially were observed in the injured hemisphere of graft recipients. The presence of intra-arterially injected cells in the host brain was confirmed by other authors. Namestnikova et al. showed that MSCs labeled with SPIO transplanted intra-arterially were detected in the basal ganglia and cerebral cortex of rat brain immediately after cell transplantation [8]. Walczak et al. observed MSCs stained with SPIO in the injured hemisphere from 2 to 24 h after intra-arterial infusion [20]. This phenomenon was also described by Janowski’s group where glial-restricted progenitors (GRPs) labeled with Molday ION infused intra-arterially were detected by MRI in the rat brain just after transplantation [17]. To prove the donor origin of cells observed in MRI, additional methods, i.e., bioluminescence or immunohistochemically, are used. Namestnikova et al. performed the co-localization studies of iron nanoparticles conjugated with fluorescence magnetic polymers MC03F and membrane marker PKH26 [21]. Walczak et al. employed the marker of proliferating cells BrdU to show MSCs infused intra-arterially in the donor tissues [20]. In our studies, to identify transplanted hBM-MSCs labeled with Molday ION in the rat brain, the co-expression of mesenchymal stem cell marker CD44 or human stem cell marker STEM121 was applied. The positively stained hBM-MSCs visible in the brain of graft recipients were localized in the vessels in the area of brain lesion during 7 days of our observation. This was also described previously by other researchers who found that hBM-MSCs infused intra-arterially were present in the brain inside blood vessels and do not migrate into parenchyma [18, 22]. There are not many studies describing EV localization in the brain after their systemic transplantation. Xin et al. observed the transfer of exosomes enriched with green fluorescence protein (GFP) from MSCs into neurons and astrocytes in the rat brain [23]. Similarly, Chen et al. visualized exosomes stained with Dil injected intra-venously in the rat brain after ischemia [12]. Recently, Lapchak et al. detected extracellular vesicles stained with DiD near the injured area of the rabbit brain after their systemic infusion [24]. Our findings are in agreement with above studies. We were able to identify EVs labeled with PKH26 in the right hemisphere of focal brain injured rats 24 h after intra-arterial transplantation.

The abilities to stimulate neurogenesis and neuroprotective properties of EVs derived from MSCs in the models of ischemic stroke have been revealed in some studies. Xin e al. showed that exosomes from MSCs promote functional recovery, induce neurite remodeling, and activate neurogenesis and angiogenesis in rats after stroke [25]. Similarly, Otero-Ortega et al. demonstrated that EVs derived from MSCs stimulate axonal growth, white matter restoration, and signal transduction and reduce motor deficits in the rats with subcortical stroke [26]. Moreover, the administration of EVs from MSCs reduces the lesion area and improves functions impaired after ischemic stroke in sheep fetuses [27]. Additionally, EVs derived from MSCs have neuroprotective effect and module peripheral immune response in a model of ischemic stroke [28]. However, the detailed role of EVs from MSCs on local immune response in the brain after ischemic stroke has not been investigated so far. After the brain injury activation of astrocytes, microglia cells and infiltration of leucocytes into the damaged tissue were detected [3, 29]. In our studies, focal brain insult induced by ouabain injection into rat striatum was accompanied with the local activation of astrocytes and microglia/macrophages and the influx of leucocytes, including T cytotoxic cells into ipsilateral hemisphere. We have noticed that transplantation of hBM-MSCs caused the decrease of the number of activated astrocytes 24 h after cell injection. It is known that MSC infusion may not only inhibit astrocytes activation but mostly promote their polarization into anti-inflammatory phenotype which leads to glutamate capture and production of neurotrophic factors [30]. In our experiments, we also observed the reduction of microglia/macrophages after intra-arterial transplantation of hBM-MSCs into rats with focal brain injury. Previously, the inhibition of microglial cells or promotion of their polarization due to MSC presence has been detected by other researchers during in vitro studies [31, 32]. Our in vivo experiments revealed that intra-arterial EV transplantation into focal brain injury rats diminishes the number of microglia/macrophages evoked by the insult. The effect was much stronger after extracellular vesicle infusion than after transplantation of their counterparts. Similar to our findings, Ruppert et al. demonstrated that extracellular vesicles derived from MSCs reduced the number of microglia pro-inflammatory cells in spinal cord injury [33]. Activation of astrocytes and microglia caused by brain injury is often accompanied with the influx of leucocytes from the periphery. In our studies, hBM-MSC transplantation led to the inhibition of leukocyte infiltration into injured rat hemisphere. Our findings are in agreement with another study demonstrating MSC infusion in stroke mice, resulting in reduced brain leukocyte infiltration [8]. Moreover, we noticed that intra-arterial injection of EVs into focal brain injured rats caused the lower number of leucocytes visible in damaged hemisphere of graft recipients. This was in contrast to the previous study of Doeppner et al. in which EVs derived from MSCs transplanted intraventricular in focal brain ischemic mice did not diminish brain leukocyte infiltration [28]. Among different types of leucocytes, T lymphocytes play an important role in immunological defense. We noticed the decrease of T CD8^+^ cytotoxic cells after hBM-MSC or EV injection. This was in accordance with the other studies where the reduction of T cytotoxic cells after MSC infusion was found [34, 35]. The inhibitory effect of EVs in vivo has not been studied up to now. However, the induction of apoptosis in activated T cells by EVs derived from mesenchymal stem cells was demonstrated in vitro [36, 37].

Brain injury induces the release of cytokines accompanied with cellular immune response. Many studies showed that interleukin-1 produced few hours after the brain ischemic damage stimulated the production of cytokines, chemokines, and cell adhesion molecules which led to blood-barrier damage [38, 39]. Previous studies revealed that transplantation of mesenchymal stem cells caused the decrease of IL-1 production by microglia due to their inhibition and T helper cells polarization into anti-inflammatory phenotype [32, 40]. In our studies, we observed the significant decrease in the level of IL-1α and IL-1β evoked by focal brain injury after MSC or EV infusion. Ebrahim et al. showed that EVs isolated from MSCs reduced the level of interleukin-1 in a model of intrauterine adhesions [41]; however, the impact of extracellular vesicles on IL-1 production in brain-injured EV recipients has not been studied so far. Another cytokine involved in pro-inflammatory response after ischemic stroke is interleukin-6. Our studies revealed that transplantation of mesenchymal stem cells or their extracellular vesicles caused statistically significant decrease of IL-6 level in focally injured rat brain probably due to the inhibitory effect of hBM-MSCs and EVs on local immunologically effector cells, i.e., microglia cells, macrophages, and neurons. Similarly, Karlupia et al. referred that MSCs injected intra-arterially led to the decrease of IL-6 level in rat model of ischemic stroke [6]. Recent studies revealed that despite of IL-1 and IL-6, different isoforms of transforming growth factors-β (TGFβ) may be efficient as pro-inflammatory factors in immune response after ischemic stroke. Xin et al. showed the increased production of TGF-β1 in the nervous tissue injured by ischemic stroke and reduced release of this cytokine after MSCs transplantation [42]. Our studies revealed significant increase of TGF-β2 production in rat brain after striatal focal injury and its significant decrease 3 and 7 days after hBM-MSC or EV infusion.

Brain injury is accompanied by high expression of chemokines that can be critical for the outcome in neurological disorders due to its widely exerted effects on cellular activity, proliferation, and survival. Silva et al. showed that ischemic stroke causes the release of chemokine C-X-C motif ligand-1 (CXCL-1) which leads to the loss of neural cells in injured tissue [43]. In our studies, we observed the elevation of CXCL-1 in the rat brain 48 h after focal injury. Intra-arterial transplantation of hBM-MSCs or EVs strongly inhibited the production of CXCL-1 in our experimental model which was probably related to the reduction of microglial/macrophages. Similar effect was observed by Donizetti-Oliveira et al.; the authors noticed that adipose tissue-derived mesenchymal stem cells (AD-MSC) injection resulted in the decrease of CXCL-1 in a model of renal disease [44]. Another chemokine reported to be released after ischemic stroke is monocyte chemattractant protein-1 (MCP-1) which induces monocyte infiltration, activates pro-inflammatory cytokines, and leads to the progression of tissue damage [45, 46]. In our studies, we observed the increase of MCP-1 in the focally injured rat brain and the statistically significant reduction of MCP-1 level after hBM-MSC or EV transplantation. This was correleted with the decrease of microglia/macrophages and astrocyte activation. Our observation was concomitant with the results of Yoo et al. who showed that injection of mesenchymal stem cells diminished MCP-1 production in a model of ischemic stroke [8]. Brain injury results also in increase of macrophage chemoattract proteins: MIP-1α and MIP-3α. It is known that macrophage chemoattract proteins produced after ischemic stroke stimulated IL-1β release and activated nitric oxide synthase which caused the further progression of tissue damage. Inhibition of MIP-3α caused reduction of the lesion in the rat brains injured by ischemic stroke [47]. Previous studies showed that MIP-1α lead to the activation of astrocytes and microglia as well as monocyte infiltration into injured tissue [48]. In our experimental model, focal striatal injury induced high level of MIP-1α and MIP-3α in the rat brain and hBM-MSC or EV injection led to reduction of MIP-1α and MIP-3α level in each time point of observation. Similar effect was referred by Sun et al.’s studies where EV transplantation in a murine model of spinal cord injury caused the decrease of MIP-1α expression [49].

As shown here, focal brain injury induced by ouabain injection into rat striatum is accompanied with the local immune reaction visible in the damaged tissue after the insult. The activation of the innate effectors and the influx of acquired immune cells from the peripheral blood are observed in the ipsilateral hemisphere of brain disorders. Intra-arterial transplantation of human bone marrow-derived mesenchymal stem cells or their extracellular vesicles infused to the internal carotid artery enables their migration into the brain. The donor cells are localized in the damaged area; however, they do not migrate into the brain parenchyma being captured inside the microvessels during 7 days of observation. The infusion of hBM-MSCs or their EVs alleviates the local immune response in rat brain evoked by the insult. The decrease of astrocyte and microglia/macrophages’ activation and the number of T lymphocytes while declining the level of pro-inflammatory cytokines and chemokines was detected. Interestingly, the suppressive effect of EVs was comparable to their counterparts, and in some situations, the reduction of immunological response in the host brain is even more efficient than observed after hBM-MSC graft.

## Conclusions

Our studies revealed that human bone marrow mesenchymal stem cells or extracellular vesicles derived from them transplanted intra-arterially modulate immune response in the brain caused by focal brain injury. In our experimental model, EVs present similar immunomodulatory properties as their cells of origin so they can be potentially used in stroke treatment instead of MSCs.

## Data Availability

The datasets used and/or analyzed during the current study are available from the corresponding author on reasonable request.

## Abbreviations

AD-MSC: Adipose tissue-derived mesenchymal stem cells
BBB: Blood-brain barrier
CCA: Common carotid artery
CNS: Central nervous system
CXCL1: Chemokine C-X-C motif ligand 1
DAMPs: Damage-associated molecular patterns
DPBS: Dulbecco’s phosphate-buffered saline
ECA: External carotid artery
EVs: Extracellular vesicles
FBS: Fetal bovine serum
GFAP: Glial fibrillary acidic protein
GFP: Green fluorescence protein
GRPs: Glial-restricted progenitors
hBM-MSCs: Human bone marrow mesenchymal stem cells
ICA: Internal carotid artery
IL-1α: Interleukin-1α
IL-1β: Interleukin-1β
IL-6: Interleukin-6
ION: Iron oxide nanoparticles
MCP-1: Monocyte chemoattractant protein-1
MIP-1α: Macrophage inflammatory protein-1α
MIP-3α: Macrophage inflammatory protein-3α
MSCs: Mesenchymal stem cells
NTA: NanoSight particle tracking analysis
PBS: Phosphate-buffered saline
RT: Room temperature
SPIO: Superparamagnetic iron oxide nanoparticles
TGF-β2: Transforming growth factor-β2
